# Determination of bacterial abundance and communities in the nipple drinking system of cascading cage layer houses

**DOI:** 10.1038/s41598-021-98330-z

**Published:** 2021-09-27

**Authors:** Yi Wan, Ruiyu Ma, Lilong Chai, Qiang Du, Rongbin Yang, Renrong Qi, Wei Liu, Junying Li, Yan Li, Kai Zhan

**Affiliations:** 1 Anhui Key Laboratory of Livestock and Poultry Product Safety Engineering, Institute of Animal Husbandry and Veterinary Medicine, Anhui Academy of Agriculture Science, Hefei, 230031 China; 2 Department of Poultry Science, University of Georgia, Athens, GA 30602 USA; 3 Anhui Sundaily Village Ecological Food Co., Ltd., Tongling, 244100 China

**Keywords:** Microbial communities, Environmental microbiology, DNA

## Abstract

Water quality is critical for egg production and animal health in commercial layer housing systems. To investigate microbial contamination in nipple drinking system in layer houses, the bacterial abundance and communities in water pipes and V-troughs on different tiers (e.g., 1st, 3rd, 5th, and 7th tiers) of a layer house with 8 overlapping cage tiers were determined using qRT-PCR and 16S rRNA sequencing. The water bacterial abundance (i.e., genome 16S rDNA copy number, WBCN) in water pipes and V-troughs did not significantly differ among tiers, but they were 46.77 to 1905.46 times higher in V-troughs than that in water pipes (*P* < 0.05) for each tier. Illumina sequencing obtained 1,746,303 effective reads from 24 water samples in V-troughs of 4 tiers (six samples from each tier). Taxonomic analysis indicated that the 1st and 5th tiers were predominated by Proteobacteria, Actinobacteria, Firmicutes and Bacteroidetes, while the 3rd and 7th tiers were predominated by Proteobacteria, Firmicutes, Bacteroidetes and Actinobacteria. The top four genera were *Acinetobacter*, *Streptococcus*, *Rothia* and *Comamonas* among measured tiers*.* The high bacterial abundance and bacterial OTUs of water in the V-troughs reflect poor water quality, which may adversely affect growth and health of laying hens. Therefore, it is suggested that water quality in the V-tough should be checked more frequently in commercial layer houses.

## Introduction

A well designed drinking water system is critical for laying hen production and animals’ health/welfare. However, poorly managed water system may serve as carriers of bacteria and lead to diseases or epidemic infections in chickens^1,2^. With the development of modern poultry production, the closed nipple drinking system has been applied on most of commercial laying hen farms that has a number of advantages as compare to open water system such as reduced labours for water check, decreased water leakage, and declined microorganism contaminations^3^. To prevent water leakage and spillage from the drinking nipples dripping to manure belts, a V-trough derived from the watering cup is usually equipped below the water pipe to collect dripping water, which is considered an efficient way to control moisture content of manure on manure belt in laying hen houses.

Nowadays, egg producers are paying more attentions to water system that is associated to both environmental quality and water quality. A range of water quality improvement products have been tested and added to disinfect drinking water in layer houses, such as oxidizers^4^, acids or oxidizers combined with organic acids^5^, and slightly acidic electrolysed water^6^. During disinfection with those disinfectants, water in the pipes need to be back-flushed for a period of time to maintain the cleaning efficiency. However, the sanitary situation in V-troughs and its potential impact on the health of chickens are overlooked in most of times. If not properly disinfected or cleaned, water in open-type V-troughs may accelerate the reproduction of bacteria such as *Escherichia coli*^7^, *Salmonella*^8^, and *Staphylococcus*^9^ due to long-term exposure to microbial aerosols indoor environment, water dripping from nipple drinkers, food scraps from chickens, and dust accumulation^10^. Unless timely and effectively managed or controlled, afformentioned bacteria may cause health issues on animals over time.

Previous studies suggested that underline feeding and drinking systems may aggravate the immune system of animals, slow down the growth of chickens, and even cause or favour the spread of infectious diseases that will eventually lead to economic loss of poultry farms^2,11^. The feeding habits of conventional caged layers or broilers indicated that chickens become accustomed to pecking and drinking the water in the V-troughs after feeding, which could have adverse effects on animal health. Therefore, it is necessary to evaluate the microbial abundance in the water of V-troughs in commercial poultry houses.

In recent years, 16S rRNA sequencing has been utilized to study and characterize bacterial community or microbiome in poultry production^12,13^. However, the microbial community of drinking water and its interactive correlation with surrounding environmental factors in poultry houses have not been studied extensively. Thus, this study aimed to investigate the bacterial abundance or communities of water in the nipple drinking system and dripping water collected by V-troughs on a commercial layer farm using 16S rRNA sequencing technology.

## Results

### The bacterial abundance in the water pipe and the V-trough

There were no significant differences in the water bacterial abundance (i.e., genome 16S rDNA copy number, WBCN) among different tiers in both the water pipe and the V-trough during different periods (Table 1). The mean value of WBCN in the water pipe on the 5th and 7th tiers was 0.16 units higher (1.45 times) than that on the 1st and 3rd tiers. The mean value of WBCN in the V-trough was the highest on the 1st tier (9.77 lg copies/mL) and the lowest on the 7th tier (9.63 lg copies/mL). By comparing the WBCNs in the water pipe and the V-trough, it was found that the mean value in the V-trough was much higher than that in the water pipe during all periods (*P* < 0.05), reaching as high as 46.77- to 1,905.46-fold greater.

**Table 1:** The bacterial abundance (WBCN, lg copies/mL) in the water pipe and the V-troughs on different tiers in the layer house during summer.

Position	Tier	Date						Mean
		06–04	06–18	07–02	07–16	07–30	08–13	
Water pipe	1st	6.54 ± 0.61	6.85 ± 0.47	6.75 ± 0.22	7.98 ± 0.27	7.02 ± 0.41	7.06 ± 0.28	7.03 ± 0.38
	3rd	6.32 ± 0.87	7.06 ± 0.35	6.91 ± 0.26	7.98 ± 0.20	7.02 ± 0.42	6.86 ± 0.13	7.02 ± 0.37
	5th	6.50 ± 0.77	6.86 ± 0.33	7.02 ± 0.17	8.12 ± 0.48	7.52 ± 0.44	7.10 ± 0.37	7.19 ± 0.42
	7th	6.39 ± 0.67	6.76 ± 0.29	7.05 ± 0.29	8.24 ± 0.33	7.61 ± 0.48	7.05 ± 0.31	7.18 ± 0.40
	Mean	6.44 ± 0.73^b^	6.89 ± 0.36^b^	6.93 ± 0.24^b^	8.08 ± 0.32^b^	7.29 ± 0.44^b^	7.02 ± 0.27^b^	
V-trough	1st	9.77 ± 0.27	9.58 ± 0.30	9.86 ± 0.25	9.76 ± 0.14	9.75 ± 0.18	9.88 ± 0.31	9.77 ± 0.24
	3rd	9.78 ± 0.22	9.71 ± 0.14	9.66 ± 0.35	9.70 ± 0.41	9.57 ± 0.47	9.84 ± 0.33	9.71 ± 0.32
	5th	9.71 ± 0.24	9.75 ± 0.12	9.76 ± 0.25	9.81 ± 0.24	9.69 ± 0.27	9.72 ± 0.51	9.74 ± 0.27
	7th	9.63 ± 0.31	9.41 ± 0.50	9.85 ± 0.25	9.73 ± 0.25	9.77 ± 0.25	9.79 ± 0.42	9.70 ± 0.33
	Mean	9.72 ± 0.26^a^	9.61 ± 0.27^a^	9.78 ± 0.27^a^	9.75 ± 0.26^a^	9.70 ± 0.29^a^	9.81 ± 0.39^a^	
Ratio of the mean WBCN in the pipe water and the mean WBCN in the V-trough water		1,905.46	524.81	707.95	46.77	257.04	616.60	

The different lowercase letters in a column indicate significant differences in values between two positions at *P* < 0.05.

### Taxonomic composition

**Figure 1:**
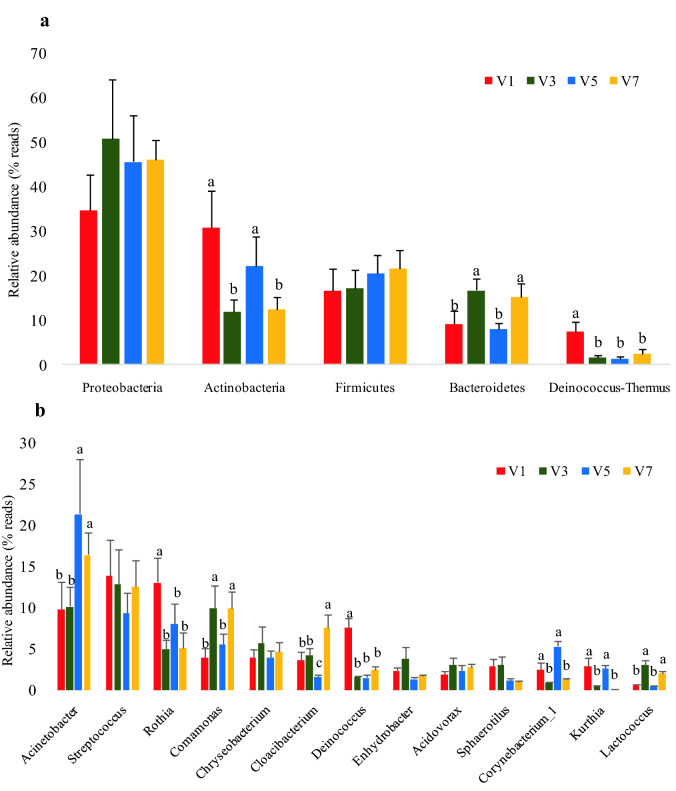
Relative abundances (% reads) of (**a**) the most dominant phyla and (**b**) the most dominant genera in the water microbial communities of different groups. Error bars represent the SDs of four group samples; boxes with a different letter above the error bars were determined to be significantly different at *P* < 0.05 by Tukey’s multiple test; V1, V3, V5, and V7 represent water samples from V-trough on the 1st, 3rd, 5th and 7th tier, respectively.

**Figure 2:**
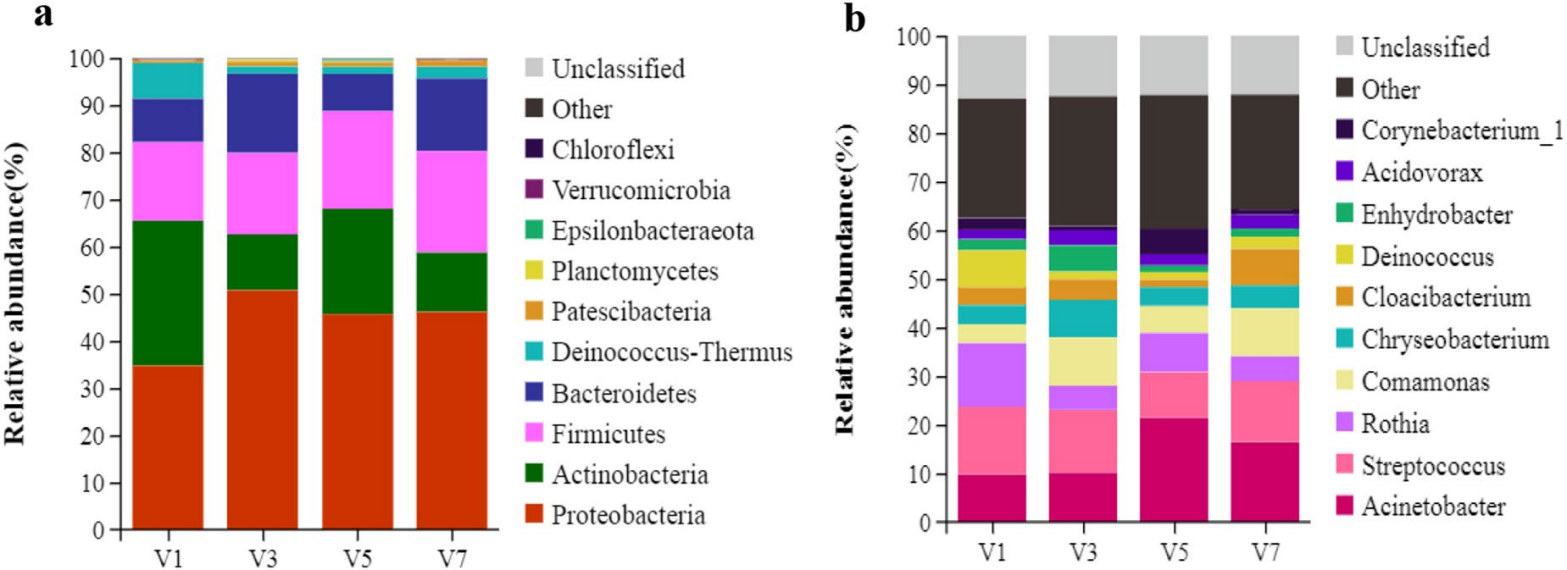
Phylum-level (**a**) and genus-level (**b**) compositions of the water microbiome in V-troughs on different tiers. A colour-coded bar plot showing the average bacterial distribution across the different tier groups that were sampled. V1, V3, V5, and V7 represent water samples from V-trough on the 1st, 3rd, 5th and 7th tier, respectively.

Based on the SILVA taxonomic database and using the analysis version of the RDP Classifier^14^, all sequences were classified from phylum to species. A total of 23 different phyla were detected in these samples. The four groups showed dissimilar taxonomic compositions at the phylum level (Figs. 1a and 2a and Supplementary Table S1). Groups V1 and V5 were predominated by Proteobacteria, Actinobacteria, Firmicutes and Bacteroidetes, while groups V3 and V7 were predominated by Proteobacteria, Firmicutes, Bacteroidetes and Actinobacteria. Detected sequences were assigned to 381 different genera. The most abundant genera (those whose relative abundances indicated that they represented more than 1% of the four libraries) among the libraries were determined to yield insights into which bacteria might be the most important (Figs. 1b and 2b and Supplementary Table S2), such as *Acinetobacter*, *Streptococcus, Rothia*, *Comamonas*, *Chryseobacterium*, *Cloacibacterium*, *Deinococcus*, *Enhydrobacter*, *Acidovorax*, *Sphaerotilus*, *Corynebacterium_1, Kurthia,* and *Lactococcus.*

### The relative abundance of bacterial communities of water samples on different tiers

Figure 1a reveals that the microbial compositions of water samples at the phylum levels differed among cage tiers. The relative abundances of Actinobacteria in V1 and V5 were significantly higher than those in V3 and V7 (*P* < 0.05), while the relative abundances of Bacteroidetes were significantly lower in groups V1 and V5 than those in V3 and V7 (*P* < 0.05). The relative abundance of Deinococcus-Thermus in V1 was significantly higher than those in the other groups (*P* < 0.05). There was little difference in the relative abundances of Proteobacteria and Firmicutes among different groups.

The relative abundances of the 13 most abundant genera in different groups are shown in Fig. 1b. The relative abundances of *Acinetobacter* in V5 and V7 were significantly higher than those in the other groups (*P* < 0.05). The relative abundances of *Rothia* and *Deinococcus* were the highest in V1 (*P* < 0.05), and the relative abundance of *Cloacibacterium* was the highest in V7 (*P* < 0.05). The relative abundances of *Corynebacterium*_1 and *Kurthia* in V1 and V5 were significantly higher than those in V3 and V7 (*P* < 0.05), while the relative abundances of *Comamonas* and *Lactococcus* in were significantly lower V1 and V5 (*P* < 0.05). There were no obvious differences in the other genera among the four groups.

## Discussion

Water drinking is one of main ways of spreading the harmful microorganisms and contaminated water could be hazardous to animals and humans humans when consumed^15,16^. Water inside the V-trough of drinking systems is exposed to the air and apt to breed bacteria that mainly originate from indoor aerosols^10^ and feed residues from oral chickens^17^. In the present study, the bacterial abundance of water in water pipe and the V-trough were successfully determined by qRT-PCR. The WBCN was much higher (1,905-fold greater at its maximum) in the V-trough than in the water pipe on each measured tier during every period in summer. This is because the water in the pipe for drinking was sterilized before it flowed into the pipes and was cyclically flushed at regular intervals in production, while the water in the V-trough was never cleaned or treated. In addition to the muddy water in the V-trough, many dirty deposits were also found in the V-trough at most monitoring sites when sampling. Therefore, bacteria in the V-trough reproduced rapidly over time, and the contamination there was greater than that in the water pipe.

Many studies have used high-throughput sequencing to investigate the in vivo or in vitro microbial diversity of poultry^13,18^. In this study, to further understand microbial contamination in the V-trough, 16S rRNA sequencing technology was used to identify the bacterial community of water in the V-troughs on different tiers. At the phylum level, the dominant bacteria in the water microbiome were Proteobacteria, followed by Actinobacteria, Firmicutes and Bacteroidetes. Proteobacteria is the largest phylum of bacteria, and its increased prevalence in the environment was reported to be a potential source of disease^19^. Representatives of Actinobacteria can be found in a diversity of habitats, and several of them are important human and animal pathogens^20^. Firmicutes species are positively correlated with the ability to harvest energy from the diet for animals and humans^21^, but increases in fecal Bacteroidetes are associated with a decrease in nutrient absorption^22^. Although the abundance of these phyla were detected in the samples, it can not demonstrate that pathogenic microorganisms present in the V-trough. In a single one phylum, there are many pathogens and probiotics, and even these two types of microorganisms can occur within one same genus. However, Firmicutes, Bacteroidetes, Proteobacteria and Actinobacteria are numerically the most dominant phyla in the gastrointestinal microbiome of chickens^23,24^, which was similar to the distribution of these dominant phyla in the water microbiome in the V-troughs, indicating some interactions between the V-trough environment and the intestinal environment. As feeding and drinking have the greatest impact on the intestinal microbiome in poultry^25^, when the water in the V-trough is drunk by chickens, microbes migrate and reproduce along the digestive system, and some of them can colonize the gut.

In the present study, the population structure of bacteria of water in the V-trough was investigated at the genus level. The top four genera (total relative abundance exceeding 30%), which varied among tiers, were *Acinetobacter*, *Streptococcus*, *Rothia* and *Comamonas*. The genus *Acinetobacter* is widespread in nature and can be obtained from water, soil and living organisms^26^. The genus *Comamonas* consists of four named species, *C. aquatica*, *C. kerstersii*, *C. terrigena* and *C. testosteroni*, that occasionally cause human and animal diseases of low virulence^27^. Although these two genera of bacteria are common environmental bacteria, the genera *Streptococcus* and *Rothia* have a wide range of tolerances to adverse growth conditions, and they are colonizers of the oral cavity that play a crucial role in the colonization of several other oral bacteria^28,29^. It cand be presumed that the *Streptococcus* and *Rothia* bacteria were spread orally in chickens, which further confirmed that chickens habitually peck and drink the water in the V-trough. In addition, other genera of bacteria with lower relative abundances were detected, such as *Chryseobacterium* and *Enhydrobacter*^30^, whose species are inhabitants of water and exhibit low virulence. The family *Deinococcaceae* is presently represented by approximately 50 species, and it has been found in diverse locations, such as sewage, animal feed and room dust^31^. Although these bacteria are not strongly associated with some epidemic diseases in chickens and they would not cause disease until certain kinds of balance was broken up, they are potential threats to the growth and health of laying hens when they accumulate to a certain level.

The similar trends in the relative abundances of some bacteria between V1 and V5 and between V3 and V7 may be related to the similar positions of these tiers; V1 and V5 were the low tiers on the first and second floors, respectively, while V3 and V7 were the high tiers. The similar WBCN and bacterial OTU values in the water from the V-trough among the four groups showed no obvious difference or regularity between cage tiers. Although the bacterial abundance and diversity were determined by sequence analysis in this study, the extracted DNA could originate from dead or dormant bacteria and therefore may not represent metabolically active bacteria. However, the high values of WBCNs and bacterial OTUs demonstrated that many bacterial microorganisms existed in V-trough water, which may be rich in multiple pathogens. Therefore, more attention should be given to the contamination of V-trough water while ensuring the safety and hygiene of drinking water inside water pipes in poultry production. The chicken farms could regularly collect water samples from the V-trough to monitor the content of bacteria and the contamination. In addition, a set of practical cleaning and technical disinfection procedures could be formulated for the V-trough and implemented in accordance with the requirements of drinking water sanitation.

To our knowledge, the current study is among the first to apply qRT-PCR and 16S rRNA gene sequencing technologies to the analysis of the bacterial abundance and communities in water from a commercial layer house with cascading cages. Our data revealed that the most dominant bacteria and the specific composition of the microbial community of the water in the V-trough did not vary obviously between cage tiers and that the bacterial abundance in the V-trough was much higher than that in the pipe, with high abundances of bacterial genome copies and OTUs, which indicated worsening microbial contamination. These novel insights provide a better understanding of pollution or contamination in the nipple drinking system and highlight important considerations for the monitoring and management of the V-trough.

## Methods

### Layer house

**Figure 3:**
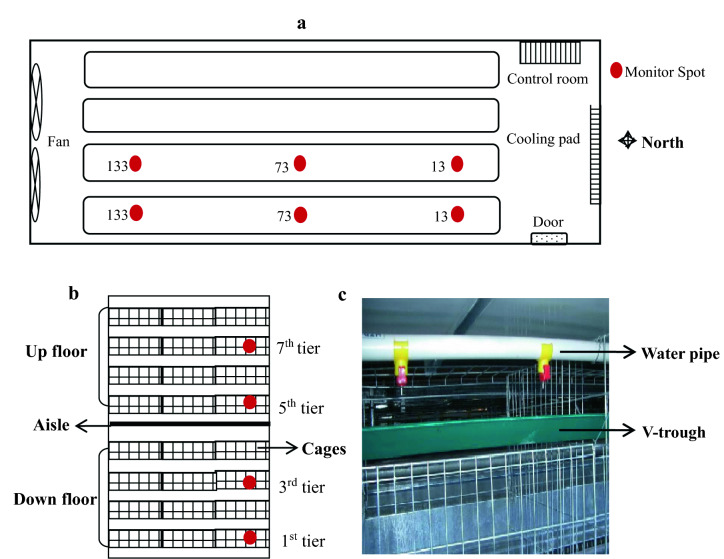
View of the layer house and diagram of the monitoring sites. (**a**) Top view of layer house; (**b**) lateral view of layer house; (**c**) the water pipe and the V-trough inside cages.

The study was approved by the Institutional Animal Care and Use Committee (IACUC) of Anhui Academy of Agricultural Science under approval number A11-CS06. All animal handling and sampling protocols were conducted according to the relevant guidelines and regulations. The experiment was conducted in an enclosed layer house with 8 overlapping tiers of cages from a commercial farm (Anhui Sundaily Village Ecological Food Co., Ltd., Tongling, China) during summer (04/06/2019 to 13/08/2019). The layer house was divided into two floors (one above the other with 4 overlapping tiers of cages on each floor) with a steel mesh aisle in the middle, and 50,000 Roman commercial laying hens were stocked. The layer house had 4 column cages and was measured 73.0 m in length, 16.0 m in width, and 6.3 m in height with a north–south major axis (Fig. 3a). There were 135 cages in a single column in a single tier. The layer house had longitudinal mechanical ventilation (exhaust fans and evaporation cooling pad) and automatic feeding, manure conveying and water lines. All chickens had free access to feed and water.

### Sampling

When the chickens were 371 days old, a total of 24 monitoring spots were selected from the front (the 13th cage), middle (the 73rd cage), and back (the 135th cage) sides on the 1st, 3rd, 5th, and 7th tiers, respectively, in the two east column cages along the longitudinal direction (Fig. 3a,b). Water samples from the water pipe at each monitoring spot were picked up directly by a screw-cap tube, while water samples inside the V-trough (Fig. 3c) were extracted by a syringe and then injected into a screw-cap tube. All water samples were collected every two weeks, and the collection volume was 50 mL. If there was insufficient water inside the V-trough, a sterile swab was used to wipe the trough and was then transferred into a screw-cap tube to which 50 mL of DEPC water was added. Samples were stored at − 4 °C until analysis. At the same time, the temperature and humidity of the monitoring spots were simultaneously recorded (Supplementary Table S3). All the sampling materials were autoclaved one day prior to the use.

### DNA extraction and PCR amplification of 16S rRNA

The collected water samples were filtered with a 0.22 µm hydrophilic PVDF (polyvinylidene fluoride) membrane. Then the filter membrane was transferred into a 2 mL centrifuge tube and was eluted with 1 mL of DEPC water. Bacterial genomic DNA was extracted from the eluate using the HiPure Water DNA Kit (Magen, Guangzhou, China) according to manufacturer’s protocols. The V4-V5 hypervariable regions of 16S rRNA were PCR-amplified from microbial genomic DNA using the following universal primers V515F (5’-GTGCCAGCMGCCGCGG-3’) and V907R (5’-CCGTCAATTCMTTTRAGTTT-3’). PCR reactions were performed in a 20 μL reaction system containing 0.8 μL of each primer, 10 ng of template DNA, 4 μL of 5 × FastPfu buffer, 2 μL of 2.5 mM dNTPs and 0.4 μL of FastPfu polymerase. Thermocycling parameters were as follows: a 2 min initial denaturation at 95 °C; 30 cycles of denaturation at 95 °C for 30 s, annealing at 61 °C for 30 s, and elongation at 72 °C for 45 s; and a final extension at 72 °C for 10 min. The amplicons were pooled, purified and then quantified using a NanoDrop 2000 UV–vis instrument (Thermo Scientific, Wilmington, DE, USA).

### Preparation of a plasmid standard

The plasmid standard for qRT-PCR (quantitative real-time polymerase chain reaction) was constructed as follows: according to the amplification position of bacterial 16S rDNA universal primers (515-F/907-R), a 412 bp sequence from positions 515–926 of *Escherichia coli* 16S rDNA was inserted into the cloning vector pUC57 (the plasmid standard in this experiment was obtained by gene synthesis), and the resulting recombinant standard plasmid was designated pUC57-16S rDNA. The total length of pUC57-16S rDNA was 3122 bp, and the extracted plasmid concentration was 142.93 ng/µL. The copy number of pUC57-16S rDNA was 4.17 × 10^10^ copies/µL. The plasmid standard was diluted to 4.17 × 10^9^ ~ 4.17 × 10^3^ copies/µL with double distilled water, and 7 concentrations were used to establish the standard curve.

### qRT-PCR

Absolute quantification with qRT-PCR was used to determine the WBCNs of samples.The qRT-PCR was performed in a final reaction volume of 20 μL containing 0.5 µL of each primer, 1 µL of template DNA, 10 µL of 2 × TB Green Premix Ex Taq II and 8 µL of double distilled water with the SYBR® Green PCR Master Mix Kit (TaKaRa, Osaka, Japan) in the CFX96 Real-Time PCR Detection System (Bio-Rad, Hercules, CA, USA) using the following protocol: a 30 s initial denaturation at 94 °C followed by 40 cycles of denaturation at 94 °C for 5 s and annealing/extension at 61 °C for 50 s. All reactions were performed in triplicate for each sample.

### 16S rRNA sequencing and bioinformatics analysis

Next-generation sequencing was performed with an Illumina HiSeq 2500 PE250 system by Gene Denovo Biotechnology Co., Ltd (Guangzhou, China) to investigate the bacterial community of water samples from V-troughs. Quality filtering and analysis of the 16S rRNA gene sequence data were performed with QIIME (V 1.7.0) as previously described^32^. The high-quality sequences were clustered into OTUs defined at 97% similarity using UPARSE (version 9.2.64) pipeline^33^. These OTUs were applied for diversity (Shannon and Simpson), richness (ACE and Chao) and rarefaction curve analyses using MOTHUR^34^. Taxonomic assignments of OTUs that reached the 97% similarity level were made using QIIME by comparison with the SILVA (http://www.arb-silva.de)^35^ databases.

**Figure 4:**
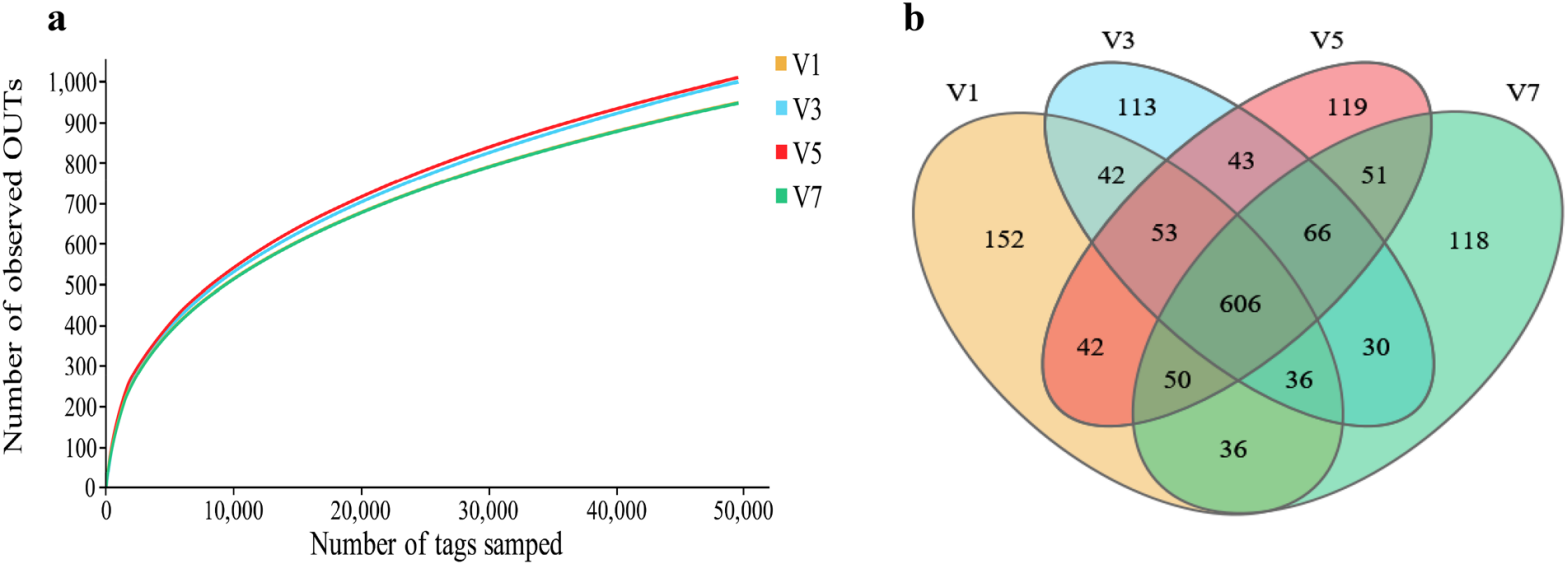
Information on operational taxonomic units (OTUs). (**a**) Rarefaction analysis of different groups. (**b**) Venn diagram showing the distribution of OTUs among different tier groups. V1, V3, V5, and V7 represent water samples from V-trough on the 1st, 3rd, 5th and 7th tier, respectively.

A total of 1,746,303 effective reads were obtained from 24 water samples (six from each of V1, V3, V5 and V7 V-troughs, which were referred to the 1st, 3rd, 5th and 7th tiers). These sequences included an average of 72,762 ± 5,985 reads per water sample, and the average length of the quality sequences was 438 ± 49 bp (Table 2). Most rarefaction curves for each sample approached a saturation plateau (Fig. 4a), which indicated that the sampling had sufficient sequence coverage to accurately describe the bacterial composition of each group. Operational taxonomic units (OTUs) were clustered at 97% sequence identity across different samples, and the average number of OTUs for each group detected by our analysis was 1,026, of which 152, 113, 119, and 118 were unique to V1, V3, V5, and V7, respectively (Fig. 4b). Bacterial richness based on OTUs was estimated by the ACE and Chao methods, and bacterial diversity was determined using the Shannon method (Table 2).

**Table 2:** Diversity estimation of the 16S rDNA gene libraries of water from the V-troughs on different tiers from sequencing analysis.

Group	Effective reads	Average length	OTU	ACE	Chao	Shannon
V1	76,443.50 ± 9142.28	434.92 ± 54.42	1027.67 ± 138.60	1483.03 ± 201.74	1483.46 ± 185.88	6.34 ± 0.61
V3	71,255.67 ± 5433.72	427.08 ± 66.69	1043.33 ± 70.51	1454.19 ± 78.79	1301.61 ± 81.52	6.08 ± 0.63
V5	70,947.50 ± 3835.73	446.83 ± 33.06	1049.50 ± 139.61	1525.86 ± 183.50	1482.53 ± 171.49	6.47 ± 0.69
V7	72,403.83 ± 3436.28	444.92 ± 40.52	984.00 ± 91.49	1398.63 ± 173.29	1371.20 ± 179.35	6.09 ± 0.54

Operational taxonomic units (OTUs) were defined at 3% dissimilarity. Richness estimators (ACE and Chao) and diversity indices (Shannon) were calculated; V1, V3, V5, and V7 represent water samples from V-trough on the 1st, 3rd, 5th and 7th tier, respectively.

### Statistical analysis

The data were subjected to analysis of variance (ANOVA) using the general linear model (GLM) command in SAS version 9.3 statistics software (SAS Institute Inc., Cary, NC, USA). Tukey’s multiple comparison was used to test the significance of the differences between group means; significance was declared at *P* < 0.05. All data are presented as the mean and standard error of the mean, and the temperature, humidity, and total copy numbers of bacterial genome 16S rDNA on the 1st, 3rd, 5th, and 7th cage tiers were the mean values from the front, middle and back side. The WBCN per 1 mL water was represented in the logarithm base 10.

## Supplementary Information

Supplementary Information.

## Data Availability

Sequences of this project have been submitted to the National Center for Biotechnology Information (NCBI) Short Read Archive (SRA) database (Accession no. SUB9998914, Bioproject: PRJNA746093).
