# Supplementary material for: Determination of bacterial abundance and communities in the nipple drinking system of cascading cage layer houses
Source: Sci Rep. 2021 Sep 27;11:19169. doi: 10.1038/s41598-021-98330-z (PMC8476603; doi:10.1038/s41598-021-98330-z)
Supplement: Supplementary file 1 — Supplementary Information. [file 41598_2021_98330_MOESM1_ESM.doc]

**Determination of bacterial abundance and communities in the nipple drinking system of cascading cage layer houses**

Yi Wan^1^, Ruiyu Ma^1^, Lilong Chai^2^, Qiang Du^3^, Rongbin Yang^3^, Renrong Qi^1^, Wei Liu^1^, Junying Li^1^, Yan Li^1^, Kai Zhan^1*^

^1^Anhui Key Laboratory of Livestock and Poultry Product Safety Engineering, Institute of Animal Husbandry and Veterinary Medicine, Anhui Academy of Agriculture Science, Hefei 230031, China

^2^Department of Poultry Science, University of Georgia, Athens, GA 30602, USA

^3^Anhui Sundaily Village Ecological Food Co., Ltd., Tongling 244100, China

***Corresponding author:** Kai Zhan, Institute of Animal Husbandry and Veterinary Medicine, Anhui Academy of Agriculture Science, Hefei 230031, China.

**Tel:** +86-0551-6216 0189

**Fax:** +86-0551-6216 0189

**E-mail:** zhankai633@126.com

**Supplementary Table S1. The relative abundance (% reads) of the most dominant phyla in the water microbial community of different groups^1^**

| Species | V1 | V3 | V5 | V7 |
| --- | --- | --- | --- | --- |
| Proteobacteria | 34.74±8.01 | 50.73±13.26 | 45.67±10.48 | 46.11±4.28 |
| Actinobacteria | 30.73±8.51^a^ | 11.85±2.87^b^ | 22.32±6.45^a^ | 12.59±2.79^b^ |
| Firmicutes | 16.61±5.05 | 17.23±4.17 | 20.65±4.17 | 21.54±4.27 |
| Bacteroidetes | 9.23±2.86^b^ | 16.79±2.50^a^ | 7.92±1.51^b^ | 15.38±2.94^a^ |
| Deinococcus-Thermus | 7.57±2.21^a^ | 1.54±0.55^b^ | 1.50±0.40^b^ | 2.48±1.08^b^ |

^1^V1, V3, V5, V7 represented water samples from V-trough on the 1^st^, 3^rd^, 5^th^ and 7^th^ tier, respectively.

^a–b^Means with different superscripts in the same row are significantly different (*P* < 0.05).

**Supplementary Table S2. The relative abundance (% reads) of the most dominant genera in the water microbial community of different groups^1^**

| Species | V1 | V3 | V5 | V7 |
| --- | --- | --- | --- | --- |
| Acinetobacter | 9.77±3.41^b^ | 10.16±2.32^b^ | 21.40±6.61^a^ | 16.42±2.64^a^ |
| Streptococcus | 13.89±4.29 | 12.86±4.23 | 9.39±2.48 | 12.58±3.15 |
| Rothia | 13.09±2.92^a^ | 4.98±1.20^b^ | 8.06±2.46^b^ | 5.05±1.98^b^ |
| Comamonas | 3.88±1.17^b^ | 9.96±2.66^a^ | 5.54±1.31^b^ | 9.88±2.13^a^ |
| Chryseobacterium | 3.92±1.03 | 5.72±1.94 | 3.88±0.91 | 4.67±1.21 |
| Cloacibacterium | 3.67±0.98^b^ | 4.22±0.93^b^ | 1.58±0.28^c^ | 7.56±1.61^a^ |
| Deinococcus | 7.57±1.21^a^ | 1.54±0.25^b^ | 1.51±0.40^b^ | 2.48±0.48^b^ |
| Enhydrobacter | 2.30±0.41 | 3.75±1.46 | 1.36±0.18 | 1.67±0.21 |
| Acidovorax | 1.93±0.32 | 3.11±0.74 | 2.30±0.71 | 2.77±0.44 |
| Sphaerotilus | 2.89±0.90 | 3.03±1.01 | 1.19±0.24 | 1.02±0.15 |
| Corynebacterium_1 | 2.45±0.92^a^ | 0.87±0.13^b^ | 5.31±0.69^a^ | 1.22±0.28^b^ |
| Kurthia | 2.96±0.89^a^ | 0.59±0.03^b^ | 2.58±0.50^a^ | 0.11±0.04^b^ |
| Lactococcus | 0.66±0.05^b^ | 3.01±0.56^a^ | 0.50±0.11^b^ | 1.99±0.29^a^ |

^1^V1, V3, V5, V7 represented water samples from V-trough on the 1^st^, 3^rd^, 5^th^ and 7^th^ tier, respectively.

^a–c^Means with different superscripts in the same row are significantly different (*P* < 0.05).

**Supplementary Table S3.** The temperature (℃) and humidity (%) on different tiers in layer house

| Date | Index | Position | | | |
| --- | --- | --- | --- | --- | --- |
|  |  | 1^st^ tier | 3^rd^ tier | 5^th^ tier | 7^th^ tier |
| 06-04 | Temperature | 29.50 | 29.55 | 30.25 | 30.35 |
|  | Humidity | 88.40 | 88.87 | 81.82 | 81.70 |
| 06-18 | Temperature | 28.95 | 29.13 | 29.48 | 29.72 |
|  | Humidity | 85.70 | 85.40 | 84.02 | 84.05 |
| 07-02 | Temperature | 28.48 | 28.77 | 29.87 | 30.08 |
|  | Humidity | 81.85 | 81.45 | 76.37 | 76.07 |
| 07-16 | Temperature | 29.52 | 29.68 | 29.73 | 29.92 |
|  | Humidity | 90.78 | 90.92 | 89.35 | 89.37 |
| 07-30 | Temperature | 30.63 | 30.82 | 31.37 | 31.48 |
|  | Humidity | 90.88 | 91.25 | 89.57 | 89.42 |
| 08-13 | Temperature | 28.92 | 29.12 | 29.88 | 30.03 |
|  | Humidity | 78.88 | 79.08 | 75.63 | 75.97 |
| Mean | Temperature | 29.33 | 29.51 | 30.10 | 30.26 |
|  | Humidity | 86.08 | 86.16 | 82.79 | 82.76 |
